# Foliar Application of Spermidine Reduced the Negative Effects of Salt Stress on Oat Seedlings

**DOI:** 10.3389/fpls.2022.846280

**Published:** 2022-04-18

**Authors:** Xia Hai, Junzhen Mi, Baoping Zhao, Biru Zhang, Zhou Zhao, Jinghui Liu

**Affiliations:** ^1^Science Innovation Team of Oats, Inner Mongolia Agricultural University, Hohhot, China; ^2^College of Life Science, Chifeng University, Chifeng, China

**Keywords:** spermidine, salt stress, oat, antioxidant defense, endogenous hormone, osmotic adjustment, ion accumulation

## Abstract

The effects of foliar application of spermidine (Spd) on the physiological aspects of salt-stressed oat seedlings were studied under greenhouse conditions. At the seedling stage, the salt-sensitive variety, namely, Caoyou 1 and the salt-tolerant variety, namely, Baiyan 2 were treated with 70 and 100 mM of salt, followed by the foliar application of 0.75 mM Spd or distilled water. Results showed that Spd application increased the activities of superoxide dismutase (SOD), peroxidase (POD), catalase (CAT), and ascorbate peroxidase (APX), and reduced the rate of O_2_^⋅–^ production and the accumulation of H_2_O_2_ and malondialdehyde (MDA). In addition, it increased the level of zeatin riboside (ZR) and the content of endogenous polyamines. The application of Spd increased the contents of soluble sugar, soluble protein, and free proline and helped maintain the osmotic balance of oat leaves. At the same time, foliar Spd treatment helped in maintaining the ion nutrition balance. Specifically, it reduced the content of Na^+^ and thereby stabilized the ratio of Na^+^/K^+^, Na^+^/Ca^2+^, and Na^+^/Mg^2+^. The effects of Spd application were more obvious for the salt-sensitive cultivar Caoyou 1 and under the lighter 70 mM salt stress.

## Introduction

Soil salinity is one of the main abiotic stresses that constitute a serious threat to field crops production in many parts of the world, especially in the arid and semiarid regions (Farnaz et al., 2017). Salinity can lead to water stress, ions accumulation, plant malnutrition, secondary metabolic disorder, and reactive oxygen species (ROS) accumulation in plants, thereby inhibiting growth and reducing yield (Zhu, 2002; Apel and Hirt, 2004; Munns and Tester, 2008; Tuteja et al., 2009). Therefore, it is important to develop methods to relieve the adverse impact of salt stress and increase crop production under salt stress. The application of exogenous substances is one of the simplest and most effective approaches (Zhu and Gong, 2013).

Polyamines (PAs) are a class of low-molecular-weight aliphatic nitrogen-containing compounds with high biological activities. They play important roles in the growth and development of plants, particularly, in improving the tolerance to adverse growing conditions. Putrescine (Put), spermidine (Spd), and spermine (Spm) are the most common PAs in plants. Among them, Spd was shown to be effective in enhancing plant resistance to stresses (Yamamoto et al., 2016; Wu et al., 2018). It can be used not only as a stress protection substance but also as a signal molecule in stress signal transduction to facilitate the construction of stress resistance mechanisms (Kasinathan and Wingler, 2004; Wen et al., 2008; Ding et al., 2009). It was reported that the application of Spd effectively enhanced antioxidant enzyme activities and reduced oxidative damage caused by salt stress in cucumber (Wu et al., 2018). Yamamoto et al. (2016) investigated the effects of polyamine biosynthesis inhibitors on the salt stress response of rice seedlings and found that decreases in polyamine biosynthesis and/or polyamine content lead to increased salt stress symptoms. In contrast, the application of exogenous Spd led to decreased Na^+^ content in the leaves of citrus seedlings and reduced salinity stress symptoms (Khoshbakht et al., 2017). The effectiveness of applying exogenous Spd on salt stress relief was found to depend not only on the species, developmental stage, application concentration, and treatment duration but also on the intensity of the stress (Parvin et al., 2014; Liu et al., 2016; Yamamoto et al., 2016; ElSayed et al., 2018).

Oat is widely cultivated all over the world, particularly in the colder regions, and is one of the most important crops for both grain and feed for its superior nutritional and health values compared with other grains. China is one of the most important producers of naked oat. At present, the annual planting area in China is about 700,000 ha, mainly distributed in the provinces of Inner Mongolia, Hebei, Shanxi, and Gansu, with Inner Mongolia having the largest acreage. In the ecotone of agriculture and pastureland in Inner Mongolia, a large area of oat acreage is threatened by salt stress. It is of great significance to understand the physiological mechanism of oat resistance to salt stress and thereby to develop a strategy for salt stress management. Previous studies have shown that maintaining a certain level of polyamines in oats is of great significance for oats to adapt to saline environments (Yan et al., 2018). This study was set forth to investigate whether the foliar application of Spd can alleviate the damage of oat seedlings under salt stress, and if so, its physiological mechanism.

## Materials and Methods

### Oat Cultivars

The oat (*Avena sativa*) cultivars used in this study were “Caoyou 1” known to be sensitive to salt stress, developed by the Ulan Chabu Academy of Agriculture and Animal Husbandry Science, and “Baiyan 2” known to be relatively tolerant to salt stress, developed by the Baicheng Academy of Agricultural Sciences, Jilin Province (Sa et al., 2014).

### Seedling Culture Conditions

Seeds of a similar size of the two oat cultivars were selected, sterilized with 4% NaClO for 10 min, rinsed with distilled water for 5–6 times, dried with filter paper, and evenly placed in a germination box, covered with the two layers of filter paper, and added with 10 ml of distilled water, and then, transferred to a thermostat for germination. The incubation conditions of the thermostat were: 16 h light (25°C) and 8 h dark (20°C), with a humidity of 80% and a canopy light intensity of 400 μmol/m^2^s. After 4 days of incubation, the seeds were transferred to a hydroponic box containing 1/2 Hoagland nutrient solution (pH 6.5 ± 0.1, the electrical conductivity of 2.0–2.2 dS/m^1^) for growth and development. The seedings were held still by a foam board placed over the hydroponic box, which had holes for the seedlings to grow through. The nutrient solution was refreshed every 2 days. The growing conditions were 16 h of light (25–30°C); 8 h of darkness (15–18°C) with supplemental light.

### Salt and Spermidine Treatment

At the two-leaf stage, the seedlings were randomly divided into 6 groups to receive the following 6 treatments:

CK: 1/2 Hoagland nutrient solution + spray of distilled water;
CS: 1/2 Hoagland nutrient solution + spray of 0.75 mM Spd;
S1: 1/2 Hoagland nutrient solution + 70 mM salt (NaCl and Na_2_SO_4_ molar ratio 1:1 mixed) + spray of distilled water;
SS1: 1/2 Hoagland nutrient solution + 70 mM salt + spray of 0.75 mM Spd;
S2: 1/2 Hoagland nutrient solution + 100 mM salt + spray of distilled water; and
SS2: 1/2 Hoagland nutrient solution + 100 mM salt + spray 0.75 mM Spd.

A stock solution (1 M) of Spd was prepared by dissolving 7.2625 g of Spd in a volumetric flask of 50 ml and the final volume was made up to the mark with distilled water. The required concentration of Spd (0.75 mM) was obtained by diluting the stock solution with distilled water. Tween-80 (0.01%) was added to the solution prior to the treatment. The control treatment was made of distilled water containing the same surfactant. The Spd and control solutions were sprayed onto the plant leaves at 6 p.m. each day for four consecutive days, and a set of physiological and biochemical indicators were determined 24 h after the last spray. Leaves from the bottom were cut and frozen immediately in liquid nitrogen and stored at −80°C before the determination of the physiological parameters. The experiment was repeated three times.

### Determination of Antioxidant Enzyme Activities

Leaf samples were taken and snap frozen in liquid nitrogen for 30 s and then stored at −80°C for subsequent analysis. Then, 0.5 g fresh leaf samples were homogenized in a phosphate buffer solution in an ice bath and then subjected to refrigerated centrifugation to obtain the crude enzyme solution for determining the superoxide dismutase (SOD) activity using the nitroblue tetrazolium (NBT) photoreduction method (Beauchamp and Fridovich, 1971). Additionally, peroxidase (POD) activity was determined using the guaiacol method (Kochba et al., 1977).

Furthermore, 0.1 g fresh leaf samples were homogenized in the phosphate buffer solution in an ice bath and then were subjected to freeze-centrifugated to obtain crude enzyme solution. Catalase (CAT) was determined using the UV absorption method (Aebi, 1984).

In addition, 0.1 g of fresh leaf samples were mixed with 1 ml reagent, homogenized in an ice bath, and then freeze-centrifugated to obtain the supernatant on ice. Ascorbate peroxidase (APX) was determined using the kit XY-W-A304 (Shanghai Youxuan Biological Technology Co., Ltd.).

### Determination of O_2_^⋅–^ Production Rate, H_2_O_2_, and Malondialdehyde (MDA) Content

To determine the O_2_^⋅–^ production rate, 0.2 g of fresh leaf samples were taken and ground and homogenized in an ice bath and freeze-centrifugated. Then, the supernatant was used to determine the O_2_^⋅–^ production rate using the hydroxylamine oxidation method (Elstner and Heupel, 1976).

In addition, 0.1 g of fresh leaf samples were taken, homogenized in an ice bath, and then freeze-centrifugated. The supernatant was taken on ice to determine the H_2_O_2_ concentration using the kit XY-W-A400 (Shanghai Youxuan Biological Technology Co., Ltd.).

A total of 0.3 g of fresh leaves were taken, ground into a homogenate in an ice bath, and MDA content was determined using the thiobarbituric acid (TBA) method (Wang et al., 2010).

### Determination of Soluble Protein, Soluble Sugar, and Proline Content

For this, 0.5 g of fresh leaves were taken and ground into a homogenate in an ice bath, and the supernatant was used to determine the content of soluble protein using the Coomassie brilliant blue method (Bradford, 1976).

Leaves samples were dried in a circulation oven at 105°C for 24 h, and 50 mg dried leaves were taken, ground, and filtered, and the supernatant was used to determine soluble sugar using the anthrone colorimetric method (Yemm and Willis, 1954).

A total of 0.5 g fresh leaves were placed into a test tube, added with 2.5 ml 3% sulfosalicylic acid, and bathed for 10 min in a boiling water bath. Then, 1.0 ml of the supernatant was taken and mixed with 1.0 ml of water, 2 ml of glacial acetic acid, and 2 ml of 2.5% acid ninhydrin. The mixture solution was placed in boiling water for 30 min to allow color development. After cooling, 5 ml of toluene was added and the solution was shaken for 30 s. After the extraction was complete, the upper layer of the red toluene solution was carefully transferred into a cuvette with a pipette and the color was determined at 520 nm, using toluene as a blank control (Bates et al., 1973).

### Determination of Polyamine and Hormone Content

Putrescine, Spd, and Spm were measured as described (Zhang et al., 2008), using the Shimadzu LC-20AT high-performance liquid chromatography (HPLC) system. First, 1.0 g of fresh leaves were taken and ground into a homogenate in pre-cooled perchloric acid (PCA, 5 ml, 5% v/v), then transferred to a centrifuge tube, extracted in an ultrasonic instrument for 40 min, centrifuged at 10,000 rpm for 5 min, and the supernatant was taken. Then, 1 ml supernatant was pipetted into a 15 ml centrifuge tube, mixed with 2 ml of 2 N NaOH and then 15 μl benzoyl chloride, and the mixture was incubated at 30°C for 40 min. After the reaction was completed, 2 ml of saturated NaCl and 2 ml of anhydrous ether were added, mixed, and extracted for 1 min. After standing for layering, 1 ml of ether layer (upper layer) was taken and dried with liquid nitrogen. Then, 1.5 ml of methanol was added to dissolve the residue. The resulting solution was then filtered through a 0.45 μm micro-membrane for later use. The determination was carried out in the Inner Mongolia Animal Husbandry and Fishery Biological Experimental Research Center.

Briefly, 0.5 g fresh leaf samples were taken and frozen in liquid nitrogen, homogenized in 2 ml of 80% methanol containing 1 mM 2, 6-di-tert-butyl-4-methylphenol, and centrifuged at 3,500 × *g* for 8 min at 4°C. The supernatant was isolated using a C-18 solid phase extraction cartridge and dried under liquid nitrogen. The residues were utilized to determine IAA, ZR, GA3, and ABA concentrations based on the enzyme-linked immunosorbent assays (ELISAs) (Yang J.C. et al., 2001; Yang Y.M. et al., 2001; Zhao et al., 2006). The determination was carried out in the Crop Chemical Control Centre of China Agricultural University.

### Determination of Ion Content

The content of Na^+^, K^+^, Ca^2+^, and Mg^2+^ was detected using atomic absorption spectrophotometry with slight modifications (Miller, 1998). For this, 0.2 g fresh leaf samples were digested in nitric acid (68%) and hydrogen peroxide (30%) solution (5:2, v/v) overnight at room temperature. Then, the mixture was boiled in a microwave digestion oven until the digestion solution was clear. Next, the solution was diluted with 0.1 mM nitric acid to 50 ml, and the content of Na^+^, K^+^, Ca^2+^, and Mg^2+^ were determined at 589, 766.5, 422.7, and 285.2 nm, respectively, using an atomic absorption spectrophotometer (Shimadzu AA7000, Japan).

### Determination of Dry Weight

Dry weights were determined 48 h after the last spray. The shoots and roots from each oat plant were dried at 105°C for 30 min and then at 85°C for 48 h, and then, weighed by an electronic balance.

### Statistical Analyses

Physiological data are expressed as the mean ± standard deviation (SD) of three independent replicates, and data analyses and graphical presentation were conducted using Microsoft Excel 2016, SAS 9.4 statistical software, and GGEbiplot (Yan, 2001).

## Results

### Responses of Various Indicators to the Treatments

**FIGURE 1:**
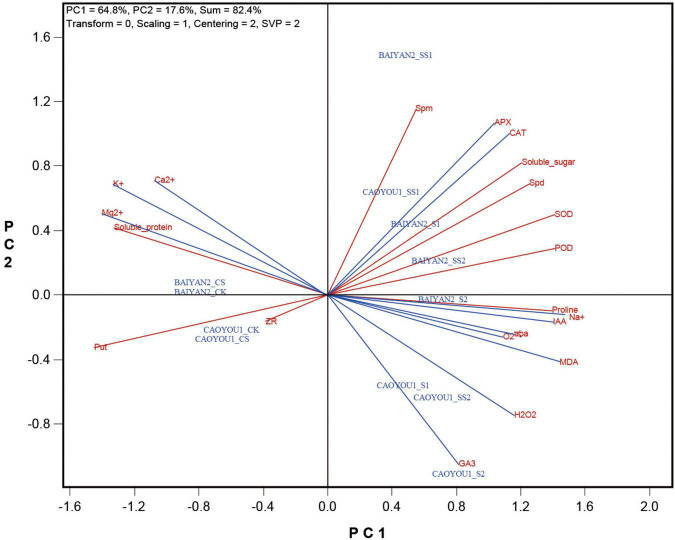
Biplot showing the relative levels of each of the cultivar-treatment combinations for each of the determined indicators.

The biplot presented in Figure 1 shows the relative values of the various indicators in each of the cultivar-treatment combinations. The biplot explained 82.4% of the total sum of squares of the variation, of which PC1 explained 64.8% and PC2 explained 17.6%. The following can be visualized from the biplot.

(1) PC1 separated the treatments into two groups: the non-stress treatments (CK and CS) for both cultivars on the left side, and the stress treatments (S1, SS1, S2, and SS2) for both cultivars on the right side of the biplot.
(2) The two cultivars under non-stress conditions showed little difference as they are placed close to each other on the biplot. They were characterized by having relatively high levels for the indicators placed on the left side (such as K^+^, Ca^2+^, Mg^2+^, put, ZR, and soluble protein), and relatively low levels for those placed on the right side (such as SOD, POD, CAT, APX, MDA, H_2_O_2_, O_2_^⋅–^, soluble sugar, proline, Spd, Spm, IAA, ABA, GA3, and Na^+^) of the biplot. The opposite can be said for the salt stress treatments. Therefore, the salt-treated and non-treated oat seedlings showed contrasting physiological states in the terms of the two sets of indicators.
(3) PC2 appeared to have separated the two cultivars under salt-stress conditions. Caoyou 1 under S2 (CAOYOU1_S2), Caoyou 1 under SS2 (CAOYOU1_SS2), and Caoyou 1 under S1 (COYOU1_S1) are placed on the upper right of the biplot, characterized by relatively high levels of GA3, H_2_O_2_, and MDA. In contrast, various treatments of Baiyan 2, particularly Baiyan 2 under SS1 are placed in the lower right of the biplot, characterized by relatively high levels of Spm, Apx, CAT, soluble sugar, etc. Caoyou 1 under SS1 (CAOYOU1_SS1) is placed on the lower right part of the biplot. This suggested that the high levels of endogenous polyamines (Spd and Spm), antioxidant enzyme activities (SOD, POD, APX, and CAT), and osmoregulation substances (proline and soluble sugar) and the low levels of ROS (O_2_^⋅–^ and H_2_O_2_) and MDA are the indicators of intrinsic or Spd-induced salt tolerance.

### The Effects of Spermidine Application on Each of the Determined Indicators

**FIGURE 2:**
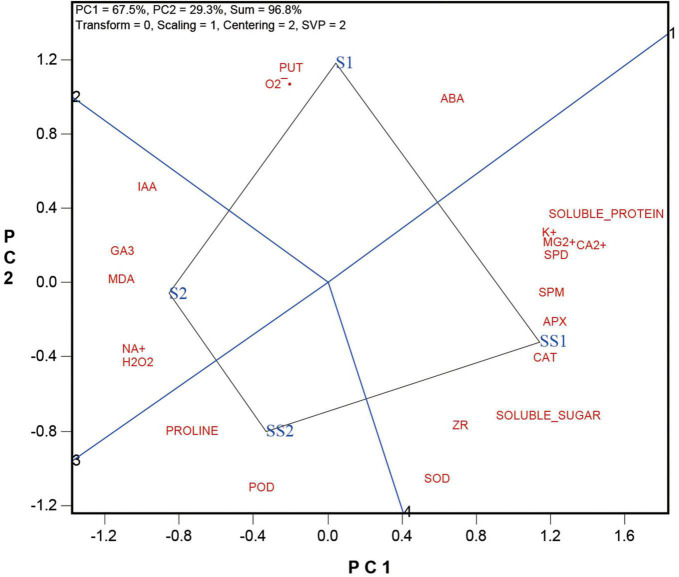
The which-won-where view of the GGE biplot to show which trait performed bets in which treatments.

The biplot presented in Figure 2 compares the four salt and Spd treatments, i.e., the two levels of salt treatments with or without Spd application (S1 vs. SS1, and S2 vs. SS2), averaged across the two cultivars. The biplot explained 96.8% of the total squares of variation, of which PC1 explained 67.5% and PC2 explained 29.3%. The following can be observed from the biplot.

(1) The polygon view of the biplot allows us to visualize which treatment had the highest level for each of the determined indicators. Specifically, it shows that S1 had the highest levels of Put, O_2_^⋅–^, and ABA, and S2 had the highest levels of IAA, GA3, MDA, Na^+^, and H_2_O_2_. SS2 had the highest levels of proline and POD; and SS1 had the highest levels of SOD, ZR, soluble sugar, CAT, APX, Spm, Ca^2+^, Mg^2+^, K^+^, and soluble protein.
(2) The distance between any two treatments is an indication of the magnitude of the difference between them. Thus, the distance between S1 and SS1 is considerably longer than that between S2 and SS2, indicating that the effect of Spd application was stronger under S1 (lower salt stress) than under S2 (higher salt stress).
(3) The straight lines labeled “1” and “3” pass the biplot origin and happen to align as a single line, which may be referred to as the Zero-Effect line. It serves the function to show the effects of the Spd application, i.e., to compare S1 vs. SS1 and S2 vs. SS2 for various indicators. SS1 and SS2 had higher levels for the indicators that are placed on their side of the Zero-Effect line but lower levels for those placed on the other side of the line. In other words, the Spd applications had stronger effects on the indicators that are placed away from the Zero-Effect line and weaker effects on those placed close to the Zero-Effect line. If an indicator is located right on the Zero-Effect line, it means that the Spd application had zero effect on its level. Thus, the biplot showed that Spd application had strong effects in increasing the levels of POD, CAT, APX, ZR, Soluble sugar, Spd, and Spm and in decreasing the levels of IAA, GA3, Na^+^, MDA, O_2_^⋅–^, and Put. These indicators will be further examined below.

### Effects of Spermidine Application on Superoxide Dismutase, Peroxidase, Catalase, and Ascorbate Peroxidase Under Salt Stress in the Oat Seedlings

**FIGURE 3:**
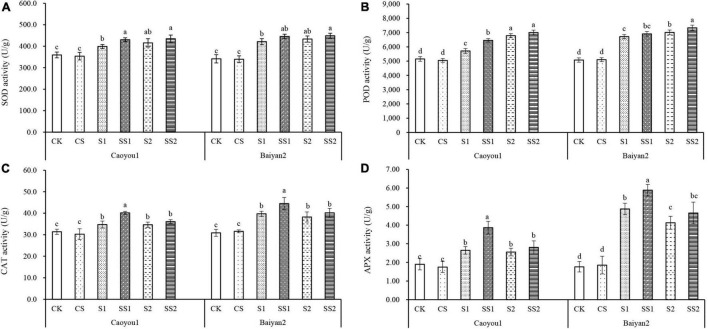
Effects of Spd application on SOD, POD, CAT, and APX under salt stress in the oat seedlings. CK, Nutrient solution + distilled water; CS, Nutrient solution + 0.75 mM Spd; S1, 70 mM salt + distilled water; SS1, 70 mM salt + 0.75 mmol/L Spd; S2, 100 mM salt + distilled water; SS2, 100 mM salt + 0.75 mmol/L Spd; Different lowercase letters indicate significant differences between treatments at 0.05 level.

For both oat cultivars, the activities of SOD, POD, CAT, and APX under S1 and S2 were significantly increased relative to CK and CS (Figure 3), these were intrinsic responses of the oat seedling to salt stress. These activities were further increased by the Spd application. Compared with S1, Spd application (SS1) led to a significant increase in SOD, POD, CAT, and APX activities, by 7.9, 12.9, 15.7, and 46.0%, respectively, for Caoyou 1. For Baiyan 2, only the CAT and APX activities were significantly increased with the Spd application by 11.9 and 20.7%, respectively. Compared with S2, Spd application (SS2) only led to an increase in the POD activity for Baiyan 2.

### Effects of Spermidine Application on O_2_^⋅–^ Production Rate, H_2_O_2_, and Malondialdehyde Content Under Salt Stress in the Oat Seedlings

**FIGURE 4:**
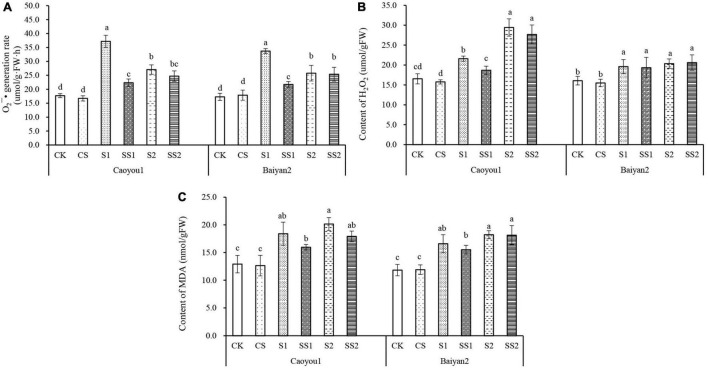
Effects of Spd application on O_2_^⋅–^ production rate, H_2_O_2_ and MDA content under salt stress in the oat seedlings. CK, Nutrient solution + distilled water; CS, Nutrient solution + 0.75 mM Spd; S1, 70 mM salt + distilled water; SS1, 70 mM salt + 0.75 mmol/L Spd; S2, 100 mM salt + distilled water; SS2, 100 mM salt + 0.75 mmol/L Spd; Different lowercase letters indicate significant differences between treatments at 0.05 level.

For both oat cultivars, the levels of O_2_^⋅–^ production rate, MDA, and H_2_O_2_ content under S1 and S2 were significantly increased relative to CK and CS (Figure 4). Compared with S1, Spd application (SS1) led to significantly decreased levels of O_2_^⋅–^ production rate and H_2_O_2_ content by 39.9 and 13.4%, respectively, for Caoyou 1. For Baiyan 2, only the O_2_^⋅–^ production rate was significantly decreased with the Spd application by 35.5%.

### Effects of Spermidine Application on Soluble Protein, Soluble Sugar, and Proline Contents Under Salt Stress in the Oat Seedlings

**FIGURE 5:**
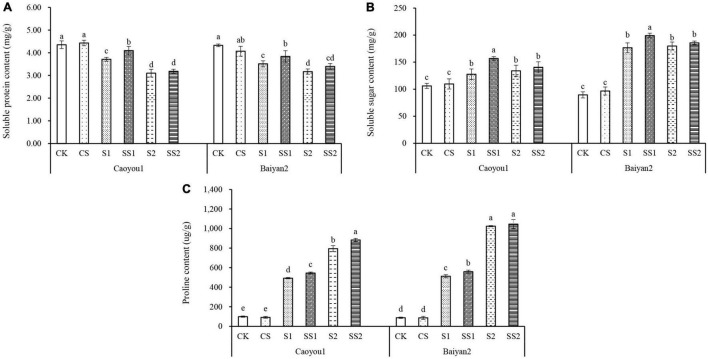
Effects of Spd application on soluble protein, soluble sugar, and proline contents under salt stress in the oat seedlings. CK, Nutrient solution + distilled water; CS, Nutrient solution + 0.75 mM Spd; S1, 70 mM salt + distilled water; SS1, 70 mM salt + 0.75 mmol/L Spd; S2, 100 mM salt + distilled water; SS2, 100 mM salt + 0.75 mmol/L Spd; Different lowercase letters indicate significant differences between treatments at 0.05 level.

Compared with CK and CS, salt stresses (S1 and S2) led to a significant increase in the levels of soluble sugar, proline, and a significant decrease in protein accumulation for both oat cultivars (Figure 5). Spd spraying further increased the levels of proline and soluble sugar under the salt stresses and alleviated the reduction in soluble protein. Compared with S1, Spd application (SS1) led to a significant increase in the levels of soluble protein, soluble sugar, and proline in the leaves of Caoyou 1 by 10.7, 22.8, and 10.3%, respectively. For Baiyan 2, the levels of soluble protein, soluble sugar, and proline were significantly increased by 9.0, 12.7, and 9.3%, respectively. Compared with S2, Spd application (SS2) only led to an increase in the proline content for the salt-sensitive cultivar Caoyou 1.

### Effects of Spermidine Application on Endogenous Polyamine Contents and Ratio Under Salt Stress in the Oat Seedlings

**FIGURE 6:**
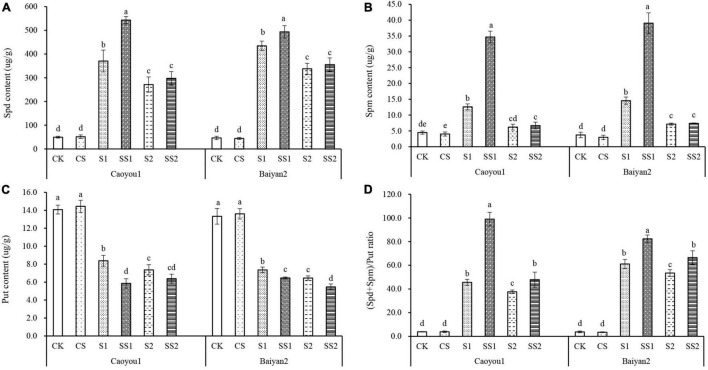
Effects of Spd application on endogenous polyamine contents and ratio under salt stress in the oat seedlings. CK, Nutrient solution + distilled water; CS, Nutrient solution + 0.75 mM Spd; S1, 70 mM salt + distilled water; SS1, 70 mM salt + 0.75 mmol/L Spd; S2, 100 mM salt + distilled water; SS2, 100 mM salt + 0.75 mmol/L Spd; Different lowercase letters indicate significant differences between treatments at 0.05 level.

Compared with CK and CS, salt stresses (S1 and S2) led to a significant increase in the levels of Spd and Spm contents and the (Spd + Spm)/Put ratio, and a significant decrease in Put content for both oat cultivars (Figure 6). Spd spraying further increased the levels of Spd and Spm contents and (Spd + Spm)/Put ratio and further decreased the Put content. Compared with S1, Spd application (SS1) led to a significant increase in the levels of Spd and Spm contents and (Spd + Spm)/Put ratio, by 46.6, 175.0, and 117.0%, respectively, and decreased the level of Put by 30.1% in the leaves of Caoyou 1. The corresponding changes for Baiyan 2 were 13.6, 168.4, 34.7, and 12.1%, respectively. Compared with S2, Spd application (SS2) only led to a significant increase in the (Spd + Spm)/Put ratio for both oat cultivars, and only to a significant decrease in the Put content for Baiyan 2.

### Effects of Spermidine Application on Endogenous Hormone Content Under Salt Stress in the Oat Seedlings

**FIGURE 7:**
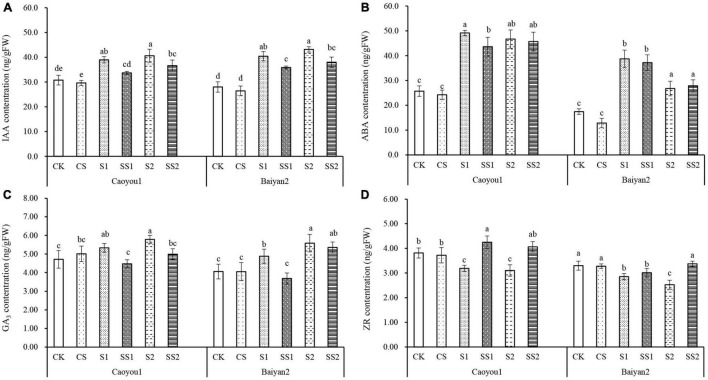
Effects of Spd application on endogenous hormone content under salt stress in the oat seedlings. CK, Nutrient solution + distilled water; CS, Nutrient solution + 0.75 mM Spd; S1, 70 mM salt + distilled water; SS1, 70 mM salt + 0.75 mmol/L Spd; S2, 100 mM salt + distilled water; SS2, 100 mM salt + 0.75 mmol/L Spd; Different lowercase letters indicate significant differences between treatments at 0.05 level.

As shown in Figure 7, compared with CK and CS, the levels of IAA, GA3, and ABA were increased under S1 and S2, and ZR decreased for both oat cultivars. Spd spraying decreased the levels of IAA, GA3, and ABA under salt stress, and increased the level of ZR. Compared with S1, Spd application (SS1) led to a significant decrease in the levels of IAA, ABA, and GA3 in leaves of Caoyou 1, by 13.5, 11.3, and 16.1%, respectively, and a significant increase in ZR by 33.2%. For Baiyan 2, the levels of IAA and GA3 were significantly decreased by 11.3 and 24.5%, respectively. Compared with S2, Spd application (SS2) led to a significant increase in IAA and GA3 in Caoyou 1 by 9.9 and 13.7%, respectively, and a significant increase in ZR by 31.0%. For Baiyan 2, the level of IAA was decreased by 12.0% and ZR increased by 33.6%.

### Effects of Spermidine Application on Ion Content and Ratio Under Salt Stress in the Oat Seedlings

**FIGURE 8:**
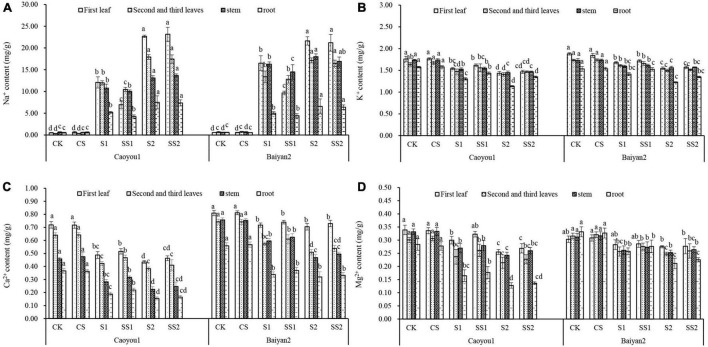
Effects of Spd application on ion content under salt stress in the oat seedlings. CK, Nutrient solution + distilled water; CS, Nutrient solution + 0.75 mM Spd; S1, 70 mM salt + distilled water; SS1, 70 mM salt + 0.75 mmol/L Spd; S2, 100 mM salt + distilled water; SS2, 100 mM salt + 0.75 mmol/L Spd; Different lowercase letters indicate significant differences between treatments at 0.05 level.

Compared with CK and CS, salt stresses (S1 and S2) led to a significant increase in Na^+^ in both oat cultivars, and the increase in leaves and stems was significantly greater than that in roots (Figure 8A). Spd spraying reduced Na^+^ under salt stress. Compared with S1, Spd application (SS1) led to a significant decrease in the level of Na^+^ in the first leaf and the second plus third leaves of Caoyou 1 by 42.4 and 12.6%, respectively. For Baiyan 2, the levels of Na^+^ in the first leaf and the stem were decreased by 41.5 and 11.0%, respectively.

Compared with CK and CS, salt stresses (S1 and S2) led to a significant decrease in the levels of K^+^ (Figure 8B). Spd spraying increased the levels of K^+^ under the salt stresses. Compared with S1, Spd application (SS1) led to a significant increase in the levels of K^+^ in the roots of Caoyou 1 by 10.0%.

Compared with CK and CS, salt stresses (S1 and S2) led to a significant decrease in the levels of Ca^2+^ (Figure 8C). Spd spraying increased the levels of Ca^2+^ under the salt stresses. Compared with S1, Spd application (SS1) led to a significant increase in the levels of Ca^2+^ in the second plus third leaves and the root of Caoyou 1 by 10.3 and 16.2%, respectively.

Compared with CK and CS, salt stresses (S1 and S2) led to a significant decrease in the levels of Mg^2+^ (Figure 8D). Spd spraying increased the levels of Mg^2+^ under the salt stresses. Compared with S1, Spd application (SS1) led to an increase in the level of Mg^2+^ in the various organs of both oat cultivars, but the changes were not statistically significant.

For both oat cultivars, the ratios of Na^+^/K^+^, Na^+^/Ca^2+^, and Na^+^/Mg^2+^ under S1 and S2 were significantly increased relative to CK and CS (Tables 1–3). Spd spraying decreased these ratios. Compared with S1, Spd application (SS1) led to a significant decrease in the ratios of Na^+^/K^+^ in the first leaf, the second and third leaves, and the stem of Caoyou 1 by 45.3, 17.3, and 8.3%, respectively. For Baiyan 2, the ratios of Na^+^/K^+^ in the first leaf and the stem were decreased by 42.8 and 12.1%, respectively. Compared with S2, Spd application (SS2) only led to a significant increase in the ratios of Na^+^/K^+^ in the second and third leaves for Caoyou 1 by 6.7% (Table 1).

**TABLE 1:** Effects of Spd application on Na^+^/K^+^ ratio under salt stress in the oat seedlings.

Variety	Organ	Ratio	Treatment					
			CK	CS	S1	SS1	S2	SS2
Caoyou 1	First leaf	Na^+^/K^+^	0.29 ± 0.05d	0.35 ± 0.11d	7.86 ± 1.01b	4.30 ± 0.37c	15.83 ± 0.45a	15.80 ± 1.01a
	Second and third leaves	Na^+^/K^+^	0.19 ± 0.05e	0.22 ± 0.03e	8.15 ± 0.59c	6.74 ± 0.41d	12.75 ± 0.32a	11.90 ± 0.57b
	stem	Na^+^/K^+^	0.37 ± 0.08d	0.36 ± 0.02d	7.07 ± 0.46b	6.48 ± 0.29c	9.07 ± 0.36a	9.31 ± 0.32a
	root	Na^+^/K^+^	0.34 ± 0.07c	0.38 ± 0.10c	3.97 ± 0.11b	2.95 ± 0.30b	6.64 ± 1.48a	6.47 ± 0.69a
Baiyan 2	First leaf	Na^+^/K^+^	0.30 ± 0.02d	0.30 ± 0.04d	9.84 ± 1.13b	5.62 ± 0.31c	13.97 ± 0.82a	13.48 ± 1.01a
	Second and third leaves	Na^+^/K^+^	0.38 ± 0.09c	0.41 ± 0.07c	8.31 ± 0.76b	7.73 ± 0.72b	11.45 ± 0.46a	10.88 ± 0.58a
	stem	Na^+^/K^+^	0.35 ± 0.04d	0.37 ± 0.09d	10.22 ± 0.47b	8.98 ± 0.89c	11.55 ± 0.48a	10.75 ± 0.69ab
	root	Na^+^/K^+^	0.39 ± 0.06c	0.36 ± 0.04c	3.55 ± 0.34b	2.92 ± 0.36b	5.36 ± 1.37a	4.74 ± 0.34a

*CK, Nutrient solution + distilled water; CS, Nutrient solution + 0.75 mM Spd; S1, 70 mM salt + distilled water; SS1, 70 mM salt + 0.75 mmol/L Spd; S2, 100 mM salt + distilled water; SS2, 100 mM salt + 0.75 mmol/L Spd. Different lowercase letters indicate significant differences between treatments at 0.05 level.*

**TABLE 2:** Effects of Spd application on Na^+^/Ca^2+^ ratio under salt stress in the oat seedlings.

Variety	Organ	Ratio	Treatment					
			CK	CS	S1	SS1	S2	SS2
Caoyou 1	First leaf	Na^+^/Ca^2+^	0.71 ± 0.09d	0.85 ± 0.26d	24.92 ± 3.88b	13.53 ± 2.04c	52.17 ± 1.11a	49.98 ± 1.70a
	Second and third leaves	Na^+^/Ca^2+^	0.49 ± 0.11a	0.57 ± 0.08a	28.36 ± 1.79b	22.50 ± 1.96c	46.89 ± 1.09d	42.86 ± 4.78d
	stem	Na^+^/Ca^2+^	1.40 ± 0.30c	1.32 ± 0.09c	38.46 ± 4.72b	31.64 ± 1.01b	58.73 ± 6.70a	56.39 ± 9.37a
	root	Na^+^/Ca^2+^	1.47 ± 0.36d	1.65 ± 0.49d	27.71 ± 3.09b	19.55 ± 2.98c	48.24 ± 6.01a	44.72 ± 3.85a
Baiyan 2	First leaf	Na^+^/Ca^2+^	0.71 ± 0.06d	0.69 ± 0.10d	23.00 ± 2.18b	13.04 ± 0.37c	30.70 ± 1.88a	29.12 ± 3.40a
	Second and third leaves	Na^+^/Ca^2+^	0.88 ± 0.21d	0.98 ± 0.20d	23.38 ± 2.89c	21.06 ± 2.56c	33.69 ± 1.17a	30.59 ± 0.25b
	stem	Na^+^/Ca^2+^	0.80 ± 0.12e	0.87 ± 0.21e	27.37 ± 1.05c	23.16 ± 1.72d	38.56 ± 3.09a	34.25 ± 3.13b
	root	Na^+^/Ca^2+^	1.07 ± 0.10c	0.98 ± 0.11c	14.87 ± 1.85b	12.03 ± 1.16b	20.40 ± 2.83a	19.33 ± 2.17a

*CK, Nutrient solution + distilled water; CS, Nutrient solution + 0.75 mM Spd; S1, 70 mM salt + distilled water; SS1, 70 mM salt + 0.75 mmol/L Spd; S2, 100 mM salt + distilled water; SS2, 100 mM salt + 0.75 mmol/L Spd. Different lowercase letters indicate significant differences between treatments at 0.05 level.*

**TABLE 3:** Effects of Spd application on Na^+^/Mg^2+^ ratio under salt stress in the oat seedlings.

Variety	Organ	Ratio	Treatment					
			CK	CS	S1	SS1	S2	SS2
Caoyou 1	First leaf	Na^+^/Mg^2+^	1.51 ± 0.17d	1.83 ± 0.63d	40.47 ± 5.79b	21.61 ± 2.95c	88.90 ± 3.57a	86.60 ± 11.81a
	Second and third leaves	Na^+^/Mg^2+^	1.05 ± 0.29d	1.19 ± 0.12d	50.90 ± 4.12b	40.41 ± 4.53c	84.43 ± 10.49a	76.99 ± 7.54a
	stem	Na^+^/Mg^2+^	1.93 ± 0.34c	1.89 ± 0.10c	40.17 ± 4.92b	36.01 ± 3.33b	54.08 ± 0.58a	52.70 ± 3.70a
	root	Na^+^/Mg^2+^	1.87 ± 0.24c	2.12 ± 0.49c	31.88 ± 4.22b	24.35 ± 3.64b	59.11 ± 13.16a	54.19 ± 4.47a
Baiyan 2	First leaf	Na^+^/Mg^2+^	1.88 ± 0.18d	1.82 ± 0.16d	58.39 ± 4.22b	33.76 ± 0.12c	78.55 ± 2.35a	77.42 ± 13.62a
	Second and third leaves	Na^+^/Mg^2+^	2.06 ± 0.48c	2.26 ± 0.43c	52.05 ± 1.96b	46.61 ± 5.16b	69.36 ± 0.96a	63.89 ± 5.28a
	stem	Na^+^/Mg^2+^	1.96 ± 0.34d	2.05 ± 0.39d	62.35 ± 2.49b	53.60 ± 9.63c	71.41 ± 5.00a	64.00 ± 1.24ab
	root	Na^+^/Mg^2+^	1.80 ± 0.24c	1.69 ± 0.05c	19.50 ± 2.20b	16.24 ± 3.15b	31.19 ± 7.77a	28.33 ± 1.36a

*CK, Nutrient solution + distilled water; CS, Nutrient solution + 0.75 mM Spd; S1, 70 mM salt + distilled water; SS1, 70 mM salt + 0.75 mmol/L Spd; S2, 100 mM salt + distilled water; SS2, 100 mM salt + 0.75 mmol/L Spd. Different lowercase letters indicate significant differences between treatments at 0.05 level.*

Compared with S1, Spd application (SS1) led to a significant decrease in the ratios of Na^+^/Ca^2+^ in the first leaf, the second and third leaves, and the root of Caoyou 1 by 45.7, 20.6, and 29.4%, respectively. For Baiyan 2, the ratios of Na^+^/Ca^2+^ in the first leaf and the stem were decreased by 43.3 and 15.4%, respectively. Compared with S2, Spd application (SS2) only led to a significant increase in the ratios of Na^+^/Ca^2+^ in the second and third leaves and the stem for Baiyan 2, by 9.2 and 11.2%, respectively (Table 2).

Compared with S1, Spd application (SS1) led to a significant decrease in the ratios of Na^+^/Mg^2+^ in the first leaf and the second and third leaves of Caoyou 1 by 46.6 and 20.6%, respectively. For Baiyan 2, the ratios of Na^+^/Mg^2+^ in the first leaf and the stem were decreased by 42.2 and 14.0%, respectively (Table 3).

### Effects of Spermidine Application on Dry Weight Under Salt Stress in the Oat Seedlings

**FIGURE 9:**
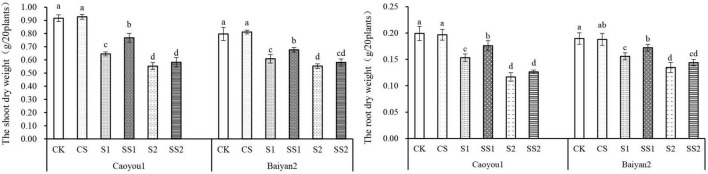
Effects of Spd application on dry weight under salt stress in the oat seedlings. CK, Nutrient solution + distilled water; CS, Nutrient solution + 0.75 mM Spd; S1, 70 mM salt + distilled water; SS1, 70 mM salt + 0.75 mmol/L Spd; S2, 100 mM salt + distilled water; SS2, 100 mM salt + 0.75 mmol/L Spd; Different lowercase letters indicate significant differences between treatments at 0.05 level.

For both oat cultivars, the shoot dry weight and root dry weight under S1 and S2 were significantly decreased relative to CK and CS (Figure 9). These dry weights were increased by the Spd application. Compared with S1, Spd application (SS1) led to a significant increase in the shoot dry weight and root dry weight by 19.0 and 14.9%, respectively, for Caoyou 1. For Baiyan 2, the shoot dry weight and root dry weight were significantly increased by the Spd application by 11.2 and 10.5%, respectively.

**FIGURE 10:**
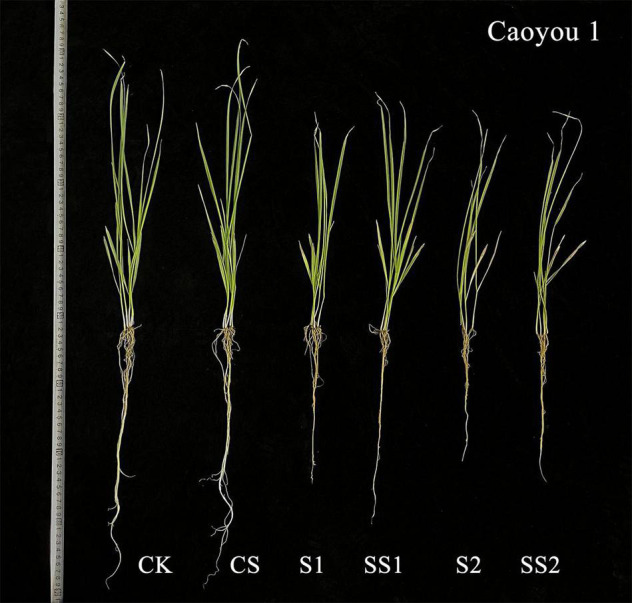
Effects of Spd application on the growth under Salt Stress in salt-sensitive cultivar Caoyou 1. CK, Nutrient solution + distilled water; CS, Nutrient solution + 0.75 mM Spd; S1, 70 mM salt + distilled water; SS1, 70 mM salt + 0.75 mmol/L Spd; S2, 100 mM salt + distilled water; SS2, 100 mM salt + 0.75 mmol/L Spd.

**FIGURE 11:**
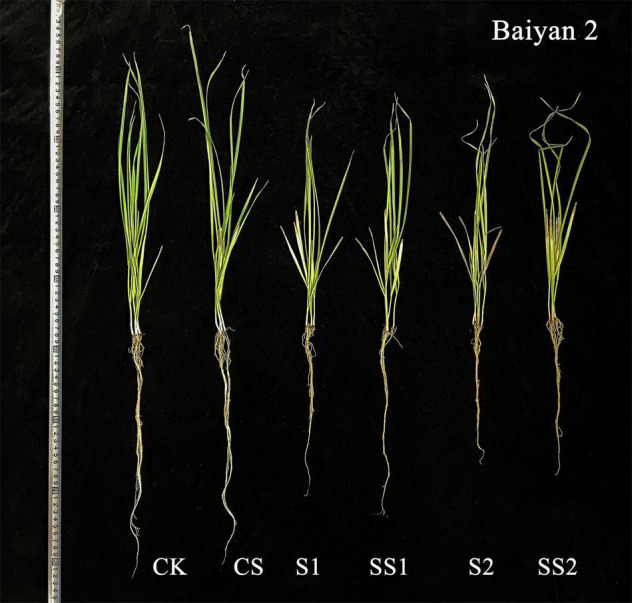
Effects of Spd application on the growth under Salt Stress in salt-tolerant cultivar Baiyan 2. CK, Nutrient solution + distilled water; CS, Nutrient solution + 0.75 mM Spd; S1, 70 mM salt + distilled water; SS1, 70 mM salt + 0.75 mmol/L Spd; S2, 100 mM salt + distilled water; SS2, 100 mM salt + 0.75 mmol/L Spd.

## Discussion

Salt stress is complex and imposes a water deficit because of osmotic effects on a wide variety of metabolic activities (Greenway and Munns, 1980; Cheeseman, 1988). This water deficit leads to the formation of ROS, such as superoxide (O_2_^⋅–^) and hydrogen peroxide (H_2_O_2_) (Halliwell and Gutteridge, 1985). These cytotoxic activated oxygen species can seriously disrupt the normal metabolism through oxidative damage to lipids (Fridovich, 1986; Wise and Naylor, 1987). MDA is one of the most important products of oxidative damage to lipids (Guidi et al., 2000). The superoxide dismutase (SOD) is the “first line of defense” of plants against oxidative damage, which helps to protect biological macromolecules from O_2_^⋅–^ attack, decompose O_2_^⋅–^ into H_2_O_2_, inhibit Haber-Weiss reaction (Chang et al., 1984), and then decompose H_2_O_2_ into H_2_O by POD, CAT, and APX, so as to prevent O_2_^⋅–^ and H_2_O_2_ accumulation (Chen and Asada, 1989; Kozi, 1992; Gill and Tuteja, 2010; Sharma et al., 2012; Sofo et al., 2015). These antioxidant enzymes work synergistically to remove excessive ROS and keep it at a low level. In the present study, the activities of SOD, POD, CAT, and APX under S1 and S2 were significantly increased relative to CK and CS for both oat cultivars (Figure 3), these were intrinsic responses of the oat seedling to salt stress. These activities were further increased by the Spd application. Compared with S1, Spd application (SS1) led to a significant increase in SOD, POD, CAT, and APX activities for both oat cultivars, and the magnitude was greater in Caoyou 1 than in Baiyan 2. For both oat cultivars, the levels of O_2_^⋅–^ production rate, MDA, and H_2_O_2_ content under S1 and S2 were significantly increased relative to CK and CS (Figure 4). Spd application reduced the accumulation of ROS. Compared with S1, Spd application (SS1) led to a significant decrease in the levels of O_2_^–^ and H_2_O_2_ content, and the magnitude of decrease in Caoyou 1 was greater than that in Baiyan 2. In general, Spd application further enhanced the activities of SOD, POD, CAT, and APX, and decreased O_2_^⋅–^ production rate, H_2_O_2_, and MDA content, consistent with the previous research results in rice (Tang et al., 2018) and wheat (ElSayed et al., 2018). The Spd-enhanced oxidant enzyme activities might originate from the characteristics of polycations, which promoted the binding with protein receptors and improved its stability. In addition, polyamine, as a signal transduction substance, could promote the synthesis of related functional proteins, thereby enhancing the activities of antioxidant enzymes in plants.

To adapt to the osmotic stress caused by salt stress, the cytoplasm accumulates low-molecular-weight compounds termed compatible solutes because they do not interfere with normal biochemical reactions (Ashihara et al., 1997; Hasegawa and Bressan, 2000); these solutes replace the water in a biochemical reaction. With accumulation proportional to the change of external osmolarity within species-specific limits, they protect the cell structures and osmotic balance and support continued water influx (or reduced efflux) (Hasegawa and Bressan, 2000). Proline (Khatkar and Kuhad, 2000; Singh et al., 2000; Lin et al., 2002), soluble sugar (Kerepesi and Galiba, 2000), and soluble protein (Bohnert and Jensen, 1996) are the most common compatible solutes. They are used as protective osmotic substances in plant stress resistance to maintain the normal function of various enzymes and cell membrane structures in plant tissues. In the present study, compared with CK and CS, salt stresses (S1 and S2) led to a significant increase in the levels of soluble sugar, proline, and a significant decrease in soluble protein for both oat cultivars (Figure 5). Spd spraying further increased the levels of proline and soluble sugar under the salt stresses and alleviated the reduction in soluble protein. Compared with S1, Spd application (SS1) led to a significant increase in the levels of soluble protein, soluble sugar, and proline, and the magnitude in Caoyou 1 was greater than that in Baiyan 2, consistent with a previous study of bermudagrass under salt stress (Shi et al., 2013). Spd application increased free proline, soluble sugar, and soluble protein in the leaves of oat seedlings under salt stress. It is speculated that Spd promoted the accumulation of proline by increasing the activities of key enzymes P5CS and P5CR in the glutamate metabolism pathway, so as to alleviate the damage of osmotic stress (Du et al., 2014), and the mitigation effect was stronger in salt sensitive cultivars. Spd application significantly increased the soluble protein content in oat seedling leaves under salt stress. On the one hand, Spd, as an information molecule, induced the expression of stress-responsive protein related genes in the process of signal transduction and promoted protein synthesis. On the other hand, after protein translation, Spd can covalently crosslink with the original protein in the cell. It may stabilize the structure of proteins and slow down their degradation (Kasukabe et al., 2004). Exogenous Spd increased soluble sugar content in oat seedlings under salt stress, probably through enhanced carbohydrate metabolism and the activities of glucose, fructose, and sucrose synthase in plants under stress (Yan et al., 2012).

Spermidine has the characteristics of polycation. It promotes the synthesis of nucleic acids and proteins by combining amino groups with negatively charged phospholipid heads and other negatively charged groups on the membrane and plays an important role in the stability of cell membrane structure (Das and Misra, 2004). Previous studies have shown that after the combination of free PAs and cinnamic acid, it is transformed into acid soluble bound PAs, which can improve the resistance of tobacco plants to salt, drought, and fungal wilting (Waie and Rajam, 2003), remove superoxide anion, and protect cell homeostasis. Increasing evidence shows that PAs plays an important role in protecting plants from abiotic stresses (Bouchereau et al., 1999). In the present study, compared with CK and CS, salt stresses (S1 and S2) led to a significant increase in the levels of internal Spd and Spm contents and (Spd + Spm)/Put ratio and a significant decrease in Put content for both oat cultivars (Figure 6). Spd spraying further increased the levels of Spd and Spm contents and (Spd + Spm)/Put ratio and further decreased Put content. Compared with S1, Spd application (SS1) led to a significant increase in the levels of Spd and Spm contents and (Spd + Spm)/Put ratio and decreased the level of Put, and the magnitude of change for the salt-sensitive cultivar Caoyou 1 was greater than that for the salt-tolerant cultivar Baiyan 2. This supports the suggestion that there may be a relationship between salt stress tolerance and endogenous PAs level (Li et al., 2014). Spd application decreased the content of Put under salt stress and increased the content of Spd and Spm, thereby increasing the (Spd + Spm)/Put ratio. A higher (Spd + Spm)/Put ratio has been shown to be beneficial to plants to offset abiotic stresses (Liu et al., 2015). Therefore, Spd application can change the levels of PAs, enhance salt stress tolerance, and protect plants from oxidative damage caused by salt stress (Bouchereau et al., 1999).

Plant endogenous hormones are important growth regulators. IAA, GA3, and ZR mainly promote growth, while ABA mainly inhibits growth (Waśkiewicz et al., 2013). ABA has long been considered as a major plant stress hormone, regulating multiple mechanisms to enhance stress tolerance (Cutler et al., 2010; Danquah et al., 2014). In this study, salt stress increased ABA concentration, which may result in stomatal closure and decreased transpiration. Spd application under salt stress decreased ABA concentration, probably because Spd alleviated injury due to the salt stress and allowed more photosynthesis. The equilibrium between zeatin nucleoside (ZR) and ABA affects the stomatal opening and closing, thus playing an important role in stress adaptation (Argueso et al., 2010; Cutler et al., 2010). In general, ZR can delay the cell senescence process by ensuring the integrity of the vacuolar membrane (Thomson et al., 1987) and promoting photosynthesis (Novakova et al., 2007). Under osmotic stress, transgenic tobacco plants with enhanced endogenous ZR levels showed the increased expression levels of photosynthetic genes associated with PSI, PSII, and cytochrome B6F (Cytb6f) complexes (Rivero et al., 2009, 2010). Cortleven et al. (2014) proved that *Arabidopsis* mutants with reduced endogenous ZR production levels are more vulnerable to photoinhibition induced by strong light stress, because the D1 protein level was significantly reduced, and the efficiency of photoprotective mechanisms, such as non-enzymatic and enzymatic clearance systems, was significantly reduced. In this study, salt stress decreased ZR concentration, while Spd application partially reversed this process. Transgenic *Arabidopsis* plants with enhanced endogenous IAA levels showed increased ETR and QP values under osmotic stress (Tognetti et al., 2010). Compared with the wild type, transgenic rice showed larger stomatal aperture and earlier wilting, which was related to the significant reduction of IAA level (Du et al., 2013). It is reported that IAA concentration in cereals and other crops increases and growth is inhibited under salt stress (Javid et al., 2011). In this study, salt stress increased the concentration of IAA, while Spd application decreased the content of IAA under salt stress. Gibberellin (GA) interacts with other hormones to jointly regulate plant growth and development under salt stress (Achard et al., 2006). Transgenic *Citrus sinensis* × *Poncirus trifoliata* plants with high endogenous GA3 levels showed significant upregulation of many genes involved in photosynthesis and stress mitigation (Huerta et al., 2010). In this study, under salt stress, Spd application reduced GA3 concentration and increased ZR concentration. Li et al. (2016) found that exogenous Spd pretreatment increased the concentration of GA3 and ZR in white clover treated by osmotic stresses. Spd application increased ZR and decreased IAA and GA_3_ which led to delayed senescence, maintained photosynthetic efficiency, and promoted plant growth under stress.

Maintaining the ion balance in plants and cells plays an important role in the normal growth and development of plants. Plants absorbed more Na^+^ and less K^+^, Ca^2+^, and Mg^2+^ under salt stress (Zhu, 2001; Bressan et al., 2008). Ca^2+^ is the main regulator of plant metabolism and development. The high concentration of Na^+^ can replace Ca^2+^ to bind on the plasma membrane and cell intima and destroy Ca^2+^ balance and increase the membrane permeability. With the decrease of K^+^ absorption, salt stress further affects Mg^2+^ absorption. Therefore, maintaining the low levels of Na^+^/K^+^, Na^+^/Ca^2+^, and Na^+^/Mg^2+^ in leaves under salt and alkali stress is an important indicator of salt and alkali tolerance. The results showed that exogenous Spd significantly reduced the content of Na^+^ in the leaves, increased the content of K^+^ in the roots, and the content of Ca^2+^ in leaves, roots, and stems, and maintained the low levels of Na^+^/K^+^, Na^+^/Ca^2+^, and Na^+^/Mg^2+^ in various organs under salt stress (Figure 8). Zhao et al. (2007) found that exogenous Spd limited Na^+^ inflow in bare roots and improved K^+^/Na^+^ homeostasis in barley seedlings by preventing K^+^ loss from stems. Other studies have shown that exogenous Spd can improve the activity of H^+^-ATPase and Na^+^/H^+^ reverse steering protein in vacuole membrane and plasma membrane of barley roots (Zhao and Qin, 2004; Mostofa et al., 2014), and help to maintain the integrity of vacuole membrane and plasma membrane under salt stress. Combined with previous studies, this study suggests that exogenous Spd improved crop salt tolerance by regulating the absorption and local distribution of ions in plants, which may be related to improvement in the activities of H^+^-PPase and Na^+^/H^+^ reversal protein in vacuole membrane and plasma membrane.

## Data Availability Statement

The original contributions presented in the study are included in the article/supplementary material, further inquiries can be directed to the corresponding author.

## Author Contributions

XH and JL designed the experiments. XH conducted the experiments and drafted the manuscript. BiZ participated in the experiments and helped in taking samples involved in this experiment. BaZ, JM, and ZZ helped revise this manuscript. All authors reviewed the manuscript and approved the submitted version.

## Conflict of Interest

The authors declare that the research was conducted in the absence of any commercial or financial relationships that could be construed as a potential conflict of interest.

## Publisher’s Note

All claims expressed in this article are solely those of the authors and do not necessarily represent those of their affiliated organizations, or those of the publisher, the editors and the reviewers. Any product that may be evaluated in this article, or claim that may be made by its manufacturer, is not guaranteed or endorsed by the publisher.
